# Molecular and biochemical characterization of carbonic anhydrases of *Paracoccidioides*

**DOI:** 10.1590/1678-4685-GMB-2015-0213

**Published:** 2016-07-25

**Authors:** Mariana Vieira Tomazett, Fabiana Fonseca Zanoelo, Elisa Flávia Cardoso Bailão, Alexandre Melo Bailão, Clayton Luiz Borges, Célia Maria de Almeida Soares

**Affiliations:** 1 Laboratório de Biologia Molecular, Instituto de Ciências Biológicas, Universidade Federal de Goiás (UFG), Goiânia, GO, Brazil; 2 Laboratório de Bioquímica, Centro de Ciências Biológicas e da Saúde, Universidade Federal de Mato Grosso do Sul (UFMS), Campo Grande, MS, Brazil

**Keywords:** carbonic anhydrases, Paracoccidioides, CO_2_, gene expression, enzyme assay

## Abstract

Carbonic anhydrases (CA) belong to the family of zinc metalloenzymes that catalyze the reversible hydration of carbon dioxide to bicarbonate. In the present work, we characterized the cDNAs of four *Paracoccidioides* CAs (CA1, CA2, CA3, and CA4). In the presence of CO_2_, there was not a significant increase in fungal *ca*1, *ca*2 and *ca*4 gene expression. The *ca*1 transcript was induced during the mycelium-to-yeast transition, while *ca*2 and *ca*4 gene expression was much higher in yeast cells, when compared to mycelium and mycelium-to-yeast transition. The *ca*1 transcript was induced in yeast cells recovered directly from liver and spleen of infected mice, while transcripts for *ca*2 and *ca*4 were down-regulated. Recombinant CA1 (rCA1) and CA4 (rCA4), with 33 kDa and 32 kDa respectively, were obtained from bacteria. The enzymes rCA1 (β-class) and rCA4 (α-class) were characterized regarding pH, temperature, ions and amino acids addition influence. Both enzymes were stable at pHs 7.5-8.5 and temperatures of 30-35 °C. The enzymes were dramatically inhibited by Hg^+2^ and activated by Zn^+2^, while only rCA4 was stimulated by Fe^2+^. Among the amino acids tested (all in L configuration), arginine, lysine, tryptophan and histidine enhanced residual activity of rCA1 and rCA4.

## Introduction

Fungi belonging to the genus *Paracoccidioides* are human pathogens that cause paracoccidioidomycosis (PCM), the most prevalent systemic mycosis in Latin America. The species of this genus grow as yeast cells at 36 °C or in vertebrate tissues, and as mycelia at 23 °C or in the soil (Franco, 1987; Restrepo *et al.*, 2001). Infection occurs when a susceptible host inhales propagules of the mycelial form, which, after reaching the lungs differentiate into the yeast phase, eventually spreading to other organs and tissues (San-Blas, 1993; Bagagli *et al.*, 2006).

Carbonic anhydrases (CAs) are metalloenzymes, that catalyze the reversible hydration of CO_2_ to generate a proton and HCO_3_^-^(Supuran, 2008a). Usually, CAs are Zn^2+^ dependent, but CAs containing Cd^2+^ or Fe^2+^ ions have been also described (Lane and Morel, 2000; MacAuley *et al.*, 2009). CAs are widely found in all life kingdoms (Eukarya, Bacteria and Archaea) playing important roles in the global carbon cycle, where anaerobic microbes convert complex biomass to methane and CO_2_ (Ferry, 2013). CAs are currently divided into five evolutionarily independent phylogenetic classes: α, β, γ, δ and ζ (Elleuche and Pöggeler, 2010). The most studied group is the α class of mammals, prokaryotes, plants and fungi. The β class was identified in plants, bacteria and fungi, but not in mammals, whereas CAs-γ, which have significantly different sequences, are predominantly found in archaea (Tripp *et al.*, 2001; Supuran, 2008a). The δ and ζ classes are only found in marine diatoms (Xu *et al.*, 2008).

The first fungal CA described in *Saccharomyces cerevisiae* is a component of a non-classical pathway of protein export, named Nce103 and classified as a β-CA (Cleves *et al.*, 1996). The mutant for *nce*103 was unable to grow under ambient conditions (0.033% CO_2_). Since the phenotype of the mutant could be restored in high concentrations of CO_2_ (5% CO_2_), it was designated as "high CO_2_ requirement" (HCR) (Amoroso *et al.*, 2005). In the filamentous ascomycete *Sordaria macrospora*, four active isoforms of β-CAs class (CAs1, CAs2, CAs3) and α-CA class (CAs4) were characterized (Elleuche and Pöggeler, 2009a). Genetic analysis of mutant strains to *cas*1, *cas*2 and *cas*3 demonstrated that Cas1 and Cas2 are involved in the formation of the fruiting body and ascospores in *S. macrospora* (Elleuche and Pöggeler, 2009b).

In *Candida albicans* and *Cryptoccocus neoformans*, CAs have been related to development and virulence (Bahn and Mühlschlegel, 2006; Mogensen and Mühlschlegel, 2008). *C. neoformans* contains two β-CAs (CAN1 and CAN2), of which CAN2 is abundantly expressed in, and essential for the growth of *C. neoformans* in their natural environment, where CO_2_ concentration is limited (0.033% CO_2_; Mogensen *et al.*, 2006). *C. albicans* contains the β-CA Nce103 and at high CO_2_ concentrations, the enzyme promotes a prominent increase in the differentiation of yeast to hyphae (Bahn and Mühlschlegel, 2006; Mogensen *et al.*, 2006).

*Paracoccidioides* has four CAs (CA1, CA2, CA3 and CA4). The CA4 was identified by Costa *et al.* (2007) in their transcriptional studies. The authors suggested that during colonization of the liver tissue, the fungus uses the fatty acids biosynthesis metabolic pathway as a survival mechanism and adaptation to this niche. The transcriptional results of that study showed that CA4 was induced during the infection process, inferring that this enzyme may provide bicarbonate for the synthesis of malonyl-CoA by acetyl-CoA carboxylase. Importantly, the acetyl-CoA carboxylase enzyme and the fatty acid synthase enzyme were up-regulated during the colonization of the liver tissue, suggesting an active lipid synthesis in this host condition. Additionally, Parente *et al.* (2011) identified by proteomic analysis, that CA1 was induced during iron limitation, a condition which mimics the host environment. In this sense, the CAs can be important to the fungus in the infection establishment, acting as a putative virulence factor.

In this study, we searched the *Paracoccidioides* (ATCC MYA-826) genome (at http://www.broad.mit.edu/annotation/genome/paracoccidioides_brasiliensis) for putative *cas* genes, and evaluated their expression in the fungus phases, as well as in yeast cells recovered from infected mice. The cDNAs for CAs (*ca1* and *ca4*) were successfully expressed in *Escherichia coli* as glutathione Sepharose 4B (GST) fusion protein. The proteins were purified and enzymatic parameters were determined *in vitro*. These results provide original information of the relevance of CAs in the physiology of *Paracoccidioides*.

## Materials and Methods

### *Paracoccidioides* growth and differentiation

*Paracoccidioides* ATCC MYA-826 was used in this study. The fungus was cultivated in solid Fava-Netto's medium [1.0% (w/v) peptone, 0.5% (w/v) yeast extract, 0.3% (w/v) proteose peptone, 0.5% (w/v) beef extract, 0.5% (w/v) NaCl, 4% (w/v) glucose and 1.2% (w/v) agar, pH 7.2]. For the mycelium-to-yeast-differentiation, the fungus was first cultivated in Fava-Neto liquid medium for 18 h at 22 °C for the mycelium growth, and subsequently, the temperature was increased to 36 °C, for the yeast growth. Cells were recovered after each incubation temperature (Bastos *et al.*, 2007).

### Fungal growth in the presence of CO_2_

*Paracoccidioides* yeast cells were aerobically grown in Fava-Netto medium plus 1.2% (w/v) agar during 5 days at 36 °C. The cells were transferred to the same medium and incubated at a 5% CO_2_ concentration [LEEC incubator; ± < 0.2% (vol/vol) CO_2_ fluctuation]. Cells were used for RNA extraction.

### Mice infection with *Paracoccidioides*

Mice were infected as previously described (Parente *et al.*, 2011). Female BALB/c mice were infected intraperitoneally with 1×10^8^ yeast cells and killed on the 7^th^ and 15^th^ days after infection. Liver and spleen were removed, placed in 1 mL of water, and homogenized with a mechanical grinder. Homogenates were filtered to remove large tissue debris and rapidly frozen with liquid nitrogen to stop transcription. Filtrates was treated with Triton X-100 at 1% final concentration (v/v) for 20 min at 37 °C and the tissue suspensions were centrifuged at 500 *g* for 5 min to further remove animal tissue. The collected supernatants were centrifuged at 4000 *g* for 3 min and the pellet was processed for total RNA isolation.

### Bioinformatic analysis

The sequences coding for CA1, CA2 and CA3 were obtained from the *Paracoccidioides* genomic database at http://www.broad.mit.edu/annotation/genome/paracoccidioides_brasiliensis/MultiHome.html. The sequence coding for CA4 was obtained from a transcript database (https://dna.biomol.unb.br/Pb/), as described by Costa *et al.* (2007). The National Center for Biotechnology Information (NCBI) BLASTp algorithm was used to search in the non-redundant database for proteins with sequence similarities to the translated full-length genes and cDNAs, taking into consideration the identity, coverage and e-value of each gene. The ScanProsite algorithm (http://ca.expasy.org/tools/scanprosite/) was used to search for motifs and conserved domains in the deduced protein, and the alignment between the homologous sequences was performed by using the CLUSTAL X algorithm, as well as the motifs described by Kerry and Ferry (2000). The PSORT II algorithm (http://psort.ims.u-tokyo.ac.jp/form2.html) was used to performed prediction of cellular localization.

### Quantitative real-time PCR

Total RNA was extracted by using the Trizol Reagent (Invitrogen Carlsbad, CA, USA) according to the manufacturer's instructions (Rezende *et al.*, 2011). Total RNA was used to synthesize cDNAs by using the high capacity RNA-to-cDNA kit (Applied Biosystems, Foster City, CA, USA). The qRT-PCR analysis was performed on a StepOnePlusTM real time PCR system (Applied Biosystems, Foster City, CA) in triplicate and values were averaged. Supplementary Table S1 shows the nucleotide sequence of the sense and antisense primers. PCR thermal cycling was performed at 40 cycles of 95 °C for 15 s followed by 62 °C for 1 min. Ten picomoles of each primer and 40 ng of template cDNA in a total volume of 25 μL SYBR green PCR master mix (Applied Biosystems) were used for each experiment. A melting curve analysis confirmed a single PCR product. The constitutive gene encoding alpha tubulin was used as the endogenous control to normalized the data. The alpha tubulin gene was amplified in each set of qRT-PCR experiments and was presented as relative expression in comparison to the experimental control cells with a value of 1. A non-template control was also included. Relative expression levels of the genes of interest were calculated using the standard curve method for relative quantification. Student's *t-*test was performed for statistical comparisons. Statistical significance was accepted for *p* values of ≤ 0.05.

### Cloning cDNAs into expression vector, heterologous expression and purification of carbonic anhydrases

Oligonucleotide primers were designed based on the DNA sequences to amplify the complete cDNAs encoding predicted CAs of *Paracoccidioides*. The nucleotide sequence of the sense and antisense primers are described in Table S1. The amplification parameters were as follows: 94 °C for 2 min, followed by 30 cycles of denaturation at 94 °C for 20 s, annealing at 50 °C for 20 s, and extension at 72 °C for 2 min; final extension at 72 °C for 5 min. The PCR products were cloned into the pGEX-4T-3 expression vector (GE Healthcare). The recombinant plasmid was used to transform *E. coli* strain *pLysS* (DE3) competent cells by using the heat shock method (Sambrook and Russel, 2001). Ampicillin-resistant transformants were obtained, and plasmid DNA was analyzed by PCR and DNA sequencing. cDNAs were transformed into *E. coli*. After, the bacteria were grown in Luria-Bertani (LB) medium supplemented with 100 μg/mL of ampicillin, at 37 °C, to absorbance of 0.6 at 600 nm, and IPTG was added to a final concentration of 0.5 mM. After 16 hours incubation, at 15 °C, the bacterial cells were harvested and suspended in phosphate saline buffer (PBS) 1X. The recombinant proteins (rCAs) fused to GST were affinity purified using glutathione Sepharose 4B (GE Healthcare). The GST was cleaved by the addition of thrombin (Sigma Aldrich) at the concentration of 1 unit/μL. Running the purified molecules on a 12% sodium dodecyl sulfate-polyacrylamide gel electrophoresis (SDS-PAGE) (w/v), followed by Coomassie blue staining, determined the purity and size of the recombinant proteins.

### Determination of carbonic anhydrase activity

Carbonic anhydrase activity was measured by the electrometric methods of Wilbur and Anderson (1948) with modifications. Initially, 0.1 mL (0.1 mg) of the sample was diluted into a final volume of 3 mL of citrate-phosphate buffer (McIlvaine, 1921), pH 8.3, and the mixture was agitated on ice for 5 min. The reaction was initiated by the addition of 2 mL ice-cold CO_2_-saturated water into the reaction vessel. Change in pH from 8.3 to 7.3 was monitored and CA activity was expressed in units per mg of protein, according to the formula [(*t_0_*/ *t-*1)10] / mg protein, where *t_0_* and *t* represent the time required for the pH to change from 8.3 to 7.3 in the buffer and sample, respectively. Protein concentrations were determined by the Bradford method (Bradford, 1976), using bovine serum albumin (BSA) as standard.

### Biochemical characterization of purified carbonic anhydrases

The suitable pH value for rCAs activity was investigated in citrate-phosphate buffer (McIlvaine, 1921) in the pH range from 5.0 to 10.0. The thermal stability was determined with rCAs incubated at different temperatures (3060° C) for 1 h in pH 8.3 citrate-phosphate buffer. After this period, enzyme activity was measured in triplicate. The influence of metal ions and amino acids on rCAs activity was investigated under standard assay conditions in the presence of 1 mM of CaCl_2,_ AgNO_3_, AlCl_3,_ BaCl_2,_ MnCl_2,_ NH_4_Cl, CoCl_2,_ ZnCl_2,_ CuCl_2,_ EDTA, FeCl_2,_ HgCl_2,_ KCl and 25 μM amino acid -L configuration (Arg, His, Ile, Leu, Lys, Met, Phe, Tre, Trp, Val).

### Analysis of the purified carbonic anhydrases and in gel activity

Electrophoresis of the rCAs under non-denaturing condition was performed in polyacrylamide gel, according to Davis (1964). For the in gel activity, a 6% polyacrylamide gel was polymerized with 1% starch (w/v). The electrophoresis was performed at alkaline pH (8.9) at a temperature of 6 ºC in buffer consisting of 50 mM Tris-HCl, 36 mM glycine, under 40 mA of current. After electrophoresis, the gel was immersed in 100 mM phosphate buffer, pH 6.5, supplemented with 10 mg of 4-metillumbeliferil acetate (dissolved in small drops of acetone) and incubated at 37 °C until the appearance of bands in UV light (Eisentthal and Danson, 1995). The method described by Laemmli (1970) was used to analyze the samples under denaturing conditions.

### Mass spectrometry analysis

The mass spectrometry analysis was performed as described previously with minor modifications (Rezende *et al.*, 2011). Briefly, the rCAs were separated by one-dimensional gels, from where they were removed, washed three times with water, and subsequently treated with 100% ACN (acetonitrile) (v/v) and dried in a speed vacuum. The samples were reduced with DDT (dithiothreitol) (10 mM) and NH_4_HCO_3_ (25 mM) for 30 min, and alkylated with iodoacetamide (55 mM) and NH_4_HCO_3_ (25 mM), under dark. Supernatant was removed and the gel pieces were washed with 25 mM ammonium bicarbonate pH 8.5, by vortexing for 10 min. Later, supernatant was removed and the gel pieces were dehydrated in 100 μL of a solution containing NH_4_HCO_3_ (25 mM) and 50% ACN (v/v), vortexed for 5 min and centrifuged. After another cycle of dehydration, enzymatic digestion was performed by incubating the samples with trypsin (12.5 ng/μL) (Roche Molecular Biochemicals), followed by rehydration at 4 °C for 10 min. Supernatant was removed and NH_4_HCO_3_ (25 mM) was added following incubation at 37 °C for 16 hours. In the digested peptides, 50% ACN (v/v) and 5% TFA (trifluoroacetic acid) (v/v) were added. After, the samples were mixed for 20 min, sonicated for 5 min, and the solution was combined with the aqueous extraction described above. The samples were dried in a speed vacuum and peptides were solubilized in water (Winters *et al.*, 2008). Two microliters of each sample was delivered to a target plate, and dried at room temperature. Later, the peptide mixtures were covered with 2 μL of 10 mg/mL alphacyano-4-hydroxycinammic acid in 50% ACN (v/v), 5% TFA (v/v). Proteins were identified by MALDI-MS/MS by using SYNAPT Q-TOF (Waters-Micromass, Manchester, UK). The mass spectrometer was calibrated by using a standard calibration mixture of known peptides with an 800-4000 Da m/z range. The MS spectra and peaks were automatically acquired and fragmented in the collision cell. The obtained MS/MS spectra were processed using Masslynx 4.1v software (Waters-Micromass, Manchester, UK) and the peak lists were created by ProteinLynx Global Server 3.0v (Waters-Micromass, Manchester, UK). The resulting peptides data were submitted to MASCOT algorithm (http://www.matrixscience.com) against the GenBank database (http://www.ncbi.nlm.nih.gov).

## Results

### *Paracoccidioides* CAs classification and transcript patterns

The cDNA sequences for the four CAs (GenBank accession numbers XM_002792385.1 (CA1); XM_002796411.1 (CA2); XM_002795151.1 (CA3); EU431184.1 (CA4) were aligned. The deduced amino acid sequences of the CA1, CA2, CA3 and CA4 presented 282, 147, 255 and 301 amino acids residues, respectively. Comparisons of the predicted protein sequences allowed their classification based on conserved regions of carbonic anhydrase, as follows: CAs signature; Zn binding amino acids and highly conserved amino acid residues in CAs, possibly important for enzyme activity (Kerry and Ferry, 2000). According to the cited criteria the CAs 1, 2 and 3 were classified as β and the CA4 was classified as α CA (Figure S1).

**Figure 1:**
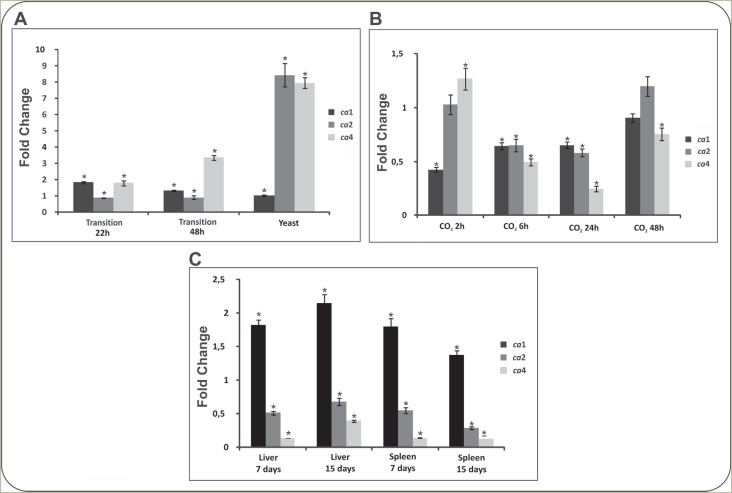
Carbonic anhydrases transcripts patterns in *Paracoccididoides*. (A) Transcripts patterns for carbonic anhydrases in phases of *Paracoccidioides.* Yeast cells were grown at 36 °C and mycelium at 22 °C. Mycelium was induced to undergo morphological differentiation for 22 and 48 hours, by changing the incubation temperature to 36 °C. The values of fold change are normalized to the mycelia phase. (B) Transcripts patterns in yeast cells incubated with 5% CO_2_. Yeast cells of *Paracoccidioides* upon incubation at 36 °C, in the presence of 5% CO_2_ at 2, 6, 24 and 48 hours. The values of fold change are normalized to the yeast cells growing *in vitro*. (C) Carbonic anhydrases transcripts patterns in yeast cells recovered from mouse liver and spleen. Expression levels of transcripts from yeast cells derived from liver and spleen of mouse after 7 and 15 days of infection. The values of fold change are normalized to yeast cells growing *in vitro*. Data of the three panels were normalized against *Paracoccidioides* alpha tubulin mRNA level. Bars indicate the standard deviation of biological triplicates and asterisks denote values statistically significant (*p* ≤ 0.05).

Figure 1A depicts transcript levels of genes encoding CAs. As observed, transcript levels were higher in *Paracoccidioides* yeast phase to *ca*2 and *ca*4, compared to mycelium. The transcript *ca*4 increased at 48 hours post-temperature shift, while *ca1* increased more significantly during the transition from mycelia to yeast at 22 hours. The *cas* expression was evaluated in yeast cells upon 5% CO_2_ for 6, 24 and 48 hours, as depicted in Figure 1B. The transcripts for the three genes, *ca1, ca2* and *ca4,* were differently regulated without correlation to the exposure time (Figure 1B). Analysis of the transcripts in yeast cells derived from infected tissues, are presented in Figure 1C. The *ca*1 expression was induced in yeast cells derived from liver and spleen of infected mice, when compared to the control. The expression level related to the *ca*3 transcript was not detected in all analyzed conditions.

### Production of recombinant carbonic anhydrases of *Paracoccidioides*

**Figure 2:**
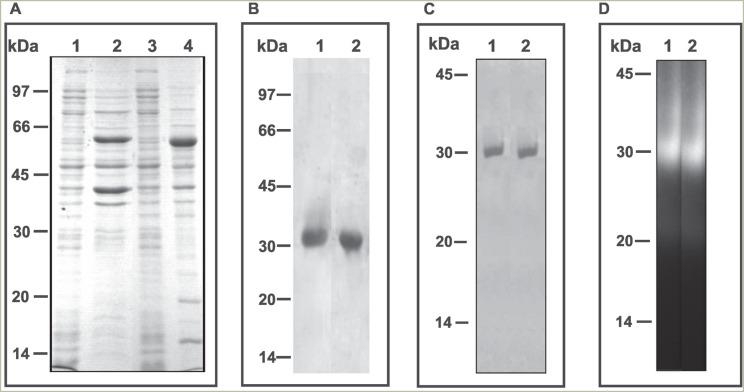
Electrophoretic profiles and in-gel activity of recombinant carbonic anhydrases of *Paracoccidioides.* The recombinant proteins (CA1 and CA4) were produced by heterologous expression. Analysis using polyacrylamide gel (SDS-PAGE 12%) was used to determine the composition of the cell lysates of *E. coli.* (A) lanes 1 and 3 are without the addition of IPTG; lanes 2 and 4 are those induced with IPTG. (B) The recombinant fusion proteins were cleaved by thrombin addition; lanes: 1 (rCA1) and 2 (rCA4). In A and B the gels were stained with Comassie Blue. (C) The purified protein samples were analyzed in native 6% PAGE stained with Comassie Blue. Lane 1 (rCA1) and lane 2 (rCA4). (D) The purified protein samples were analyzed in native PAGE and incubated with the synthetic compound 4-metillumbeliferil for carbonic anhydrases activity; lane 1 (rCA1) and lane 2 (rCA4).

We selected for further assays, the enzymes CA1 and CA4, since they represent distinct classes of CAs, β and α, respectively. The expression of recombinant proteins in *E. coli* yielded about 10 mg of the fusion protein per liter of culture. The predicted molecular sizes of the rCA1 and rCA4 were 62 and 61 kDa, respectively, considering the 29 kDa of glutathione S-transferase tag of *Schistosoma japonicum*, which were compatible with the experimentally obtained sizes, evaluated by linear regression calculations. Analysis using 12% polyacrylamide gel (SDS-PAGE) (Figure 2A) was used to determine the profile of the cell lysates of *E. coli* without the addition of IPTG, as well as those induced with IPTG. The fusion protein was purified using glutathione-sepharose 4B and the molecular size of recombinant fusion protein was the same as the predicted size. The fusion protein was cleaved by addition of thrombin protease. The purified proteins migrated in 12% SDS-PAGE as a single protein band with a molecular mass of 33 and 32 kDa for rCA1 and rCA4, respectively (Figure 2B).

### In gel activity of purified recombinant CAs

The rCA1 and rCA4 were subjected to gel analysis in non-denaturing conditions, displaying one characteristic protein band in 6% PAGE (Figure 2C), indicating that a pure protein was obtained after the final step of purification. The in gel activity was determined using polyacrylamide/starch gel (Figure 2D) in the presence of the substrate 4-metillumbeliferil and visualization in UV light. The proteins were visualized by the halo of hydrolysis (Figure 2D), coincident to the bands obtained in Figure 2C.

### Analysis by mass spectrometry (MS) of purified CAs

The rCA1 and rCA4 proteins were subjected to tryptic digestion after electrophoresis in polyacrylamide gel, and peptide maps were obtained by mass spectrometry. Results depicted in Table 1 confirmed the identity of the recombinant proteins.

**Table 1:** Analysis by mass spectrometry (MS) of the recombinant *Paracoccidioides* CAs.

CAs	GenBank Number^a^	Protein ID^b^	MASCOT SCORES				Theorical^e^ MW/p*I*
			PMF		MS/MS		
			Score^c^	Coverage (%)^d^	Score^c^	Number of sequenced peptides	
CA1	gi I 29566377	Carbonic anhydrase	124	74	73	3	32616/9.11
CA4	gi I 22567798	Carbonic anhydrase	66	63	–	–	25567/6.75

a GenBank accession numbers (http://www.ncbi.nlm.nih.gov).

b Protein name identified in Mascot.

c Score obtained from the Mascot search for each match.

d Percentage of predicted protein sequence covered by matched peptides via Mascot.

e Theoretical MW and pI calculated from amino acid sequence.

### Effect of pH, temperature, metallic ions and amino acids on activity of *Paracoccidioides* carbonic anhydrases

**Figure 3:**
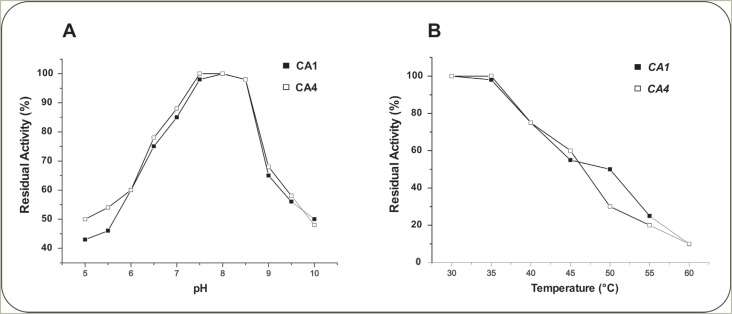
Effects of pH and temperature on stability of purified recombinant carbonic anhydrases. (A) The enzymes rCA1 and rCA4 were incubated in citrate-phosphate buffer in different pHs for 2 hours at room temperature. The residual activity was measured according to the standard enzyme assay. (B) Effect of temperature on stability of purified carbonic anhydrase (rCA1 and rCA4). The reaction mixture containing purified enzyme was incubated at different temperatures between 30 °C at 60 °C for 1 hour. The residual enzyme activity was determined.

The effect of pH on stability of rCA1 and rCA4 was examined using citrate-phosphate buffer at pH values ranging from 5.0 to 10.0. Both enzymes retain 100% activity at pH range of 7.5-8.5, after 1 hour incubation at room temperature (Figure 3A). rCA1 and rCA4 were sensitive to pH values below 7.5 and above 8.5. However, the enzymes exhibited stability even at extreme acid conditions, and approximately 43% and 45% activity was observed at pH 5.5 for rCA1 and rCA4, respectively.

Like most CA, rCA1 and rCA4 activities appear to be influenced by temperature, as demonstrated in Figure 3B. The maximum CA activity was recorded at 30-35 °C for both enzymes, while 72% and 77% activity were retained at 40 °C, for rCA1 and rCA4, respectively. In contrast, only 10% residual activity was observed at 60 °C. There was a progressive decline in enzyme activity at temperatures higher than 40 °C. Furthermore, after thermic treatment, the proteins were analyzed by PAGE under non-denaturing conditions and activity was analyzed in gel. After 30 min of heat treatment at 45 °C the in gel activity was no longer detected (data not shown).

The activity of rCA1 and rCA4 was examined under the influence of different metal ions at a concentration of 1mM (Table 2). While Hg^2+^ was a strong inhibitor for both enzymes, most of the other metals, such as Mg^2+^, Ca^2+^, Al^3+^, Co^2+^ had no effect. However, rCA1 and rCA4 were stimulated in 35% and 42%, respectively, by the addition of Zn^2+^. The rCA4 was stimulated in 38% by Fe^2+^. Some amino acids were tested for CA activity. The results presented in Table 3 show that amino acids produce similar effects on each pure enzyme. Arginine, lysine, tryptophan and histidine enhance values of residual activity of rCA1 and rCA4.

**Table 2:** Effect of metallic ions on *Paracoccidioides* carbonic anhydrase (CA) activity (CA1 and CA4).

Substance(1mM)	CA1- Residual Activity (%)	CA4- Residual Activity (%)
Control	100(± 1.72)	100(± 1.02)
CaCl_2_	104(± 2.08)	110 (± 2.33)
AgNO_3_	94(± 1.45)	97(± 2.50)
AlCl_3_	103 (± 2.72)	100(± 1.53)
BaCl_2_	102 (± 1.15)	96(± 2.65)
MnCl_2_	110 (± 1.20)	106(± 1.83)
NH_4_Cl	90 (± 1.73)	97(± 2.43)
CoCl_2_	102 (± 1.85)	98(± 1.93)
ZnCl_2_	135 (± 2.96)	142(± 1.46)
CuCl_2_	88 (± 1.45)	90(± 2.70)
EDTA	77 (± 1.35)	71(2.25)
FeCl_2_	104 (± 1.45)	138 (± 1.50)
HgCl_2_	18 (± 2.38)	15 (± 2.83)
KCl	102 (± 2.08)	100 (± 2.15)

Conditions: Enzyme activity measured in absence of any metal ion was taken as 100%. The remaining CAs activity was measured after 1 hour of incubation of purified enzyme with each metal ion.

**Table 3:** Effect of amino acids on carbonic anhydrase activity*

Compound (25M)	CA1- Residual Activity (%)	CA4- Residual Activity (%)
Control	100(± 0.82)	100(± 1.32)
L-Arg	130(± 2.00)	132(± 2.72)
L-His	142(± 2.36)	152(± 1.42)
L-Ile	110 (± 1.72)	112(± 0.48)
L-Leu	108(± 0.52)	106(± 1.75)
L-Lys	138(± 1.02)	140(± 1.64)
L-Met	106(± 2.26)	105(± 0.84)
L-Phe	100(± 2.18)	100(± 1.05)
L-Tre	100(± 0.72)	100(± 1.45)
L-Trp	162(± 2.32)	158(± 2.87)
L-Val	104(± 0.85)	104(± 1.72)

* Conditions: Enzyme activity measured in absence of any amino acids was taken as 100%. The remaining CAs activity was measured 1 hour after incubation of purified enzyme with each amino acid.

## Discussion

The current work is the first to report the identification of CAs in the genus *Paracoccidioides.* All known fungal CAs belong either to the α- or β-classes, and the genomes of most filamentous ascomycetes contain three isoforms of β-class CAs and at least one α-class CA (Elleuche and Pöggeler, 2009a). Accordingly, the genome of *Paracoccidioides* contains four genes encoding CAs, whose proteins CA1, CA2 and CA3 belong to the β-class and CA4 to the α-class.

The CAs are differentially regulated in the different phases of *Paracoccidioides*. The transcript levels of *ca2* and *ca4* were higher in the yeast phase, compared to mycelium, suggesting that these enzymes can be important for the yeast parasitic form. *C. neoformans* has two CAs, CAN1 and CAN2, of which only *CAN2* is abundantly expressed and essential for fungal growth in its CO_2_-limited natural environment (Mogensen *et al.*, 2006). *C. albicans* has only one carbonic anhydrase NCE103, which is essential for the fungus growth when it is in the host's skin in the form of yeast, and dispensable during systemic infection when transiting to a filamentous form (Klengel *et al.*, 2005). Therefore, CA could be related to fungi adaptation to microenvironments.

Transcriptional regulation of *ca* in response to CO_2_ levels has been reported in fungi. In *S. cerevisiae*, the *NCE103* gene, encoding CA is up-regulated by low CO_2_ (Amoroso *et al.*, 2005). In *S. macrospora, cas1* and *cas3* genes are differentially regulated by CO_2_ levels during ascospore germination and vegetative growth (Elleuche and Pöggeler, 2009a). In *C. albicans,* the CA functions as a CO_2_ scavenger, essential for pathogenicity in niches where available CO_2_ is limited (Klengel *et al.*, 2005). Similar to *C. albicans*, growth and morphogenesis of *C. neoformans* is strongly influenced by CO_2_, where the activity of the carbonic anhydrase CAN2 is essential to survival and proliferation as well as for basidia and basidiospore formation, but is dispensable for lethality during infection (Bahn *et al.*, 2005). In *Paracoccidioides*, yeast cells subjected to high CO_2_ concentration showed different levels of CAs expression, but without a significant increase. The role of CAs in *Paracoccidioides* cells submitted to changes in CO_2_ concentrations deserves additional studies.

The *Paracoccidioides* CAs have been described in host-mimicking conditions, as depicted in this work. Previous transcriptional data obtained by Costa *et al.* (2007) demonstrated that CA4 transcript was induced during the colonization of mice liver tissue, suggesting the relevance of this gene in the mouse infectious process. Also, the CA1 was up-regulated during iron limiting condition and CA2 was down-regulated under carbon starvation, suggesting that CAs could perform different functions according to the environment in which the pathogen is faced (Parente *et al.*, 2011; Lima *et al.*, 2014).

In *C. neoformans* and *C. albicans*, the role of CAs has been related to development and virulence (Bahn and Mühlschlegel, 2006; Mogensen and Mühlschlegel, 2008). High concentrations of CO_2_ promote filamentation in *C. albicans*, the fungus pathogenic morphology. In *C. neoformans*, bicarbonate formed through CO_2_ hydration at low concentrations by CAN2 directly activates adenylate cyclase (CAC1), which is required for capsule biosynthesis. It has been suggested that during colonization of the liver tissue by *Paracoccidioides*, the fungus can use multiple carbon sources, like glucose and products of the glyoxylate cycle. In addition, lipids biosynthesis appears to be very active, suggesting plentiful availability of carbohydrates and energy. The transcriptional results presented here, evidenced that *ca4* was induced during the infection process in liver, suggesting that the enzyme might provide bicarbonate for the synthesis of malonyl-CoA. This suggestion, however, should be experimentally addressed in the future.

Several ascomycetes CAs isoenzymes were found in cytoplasm, mitochondria or secreted (Elleuche and Pöggeler, 2010). Through *in silico* analysis using the PSORT II algorithm (http://psort.ims.u-tokyo.ac.jp/form2.html), *Paracoccidioides* CA1 was predicted to be mitochondrial, CA2 and CA3 cytoplasmic, and vesicles presumably secrete CA4 (data not shown). In *S. macrospora*, Cas 1 and Cas 3 were classified as mitochondrial and Cas 2 cytoplasmic (Elleuche and Pöggeler, 2009a).

As discussed above, CA1 and CA4 are zinc-dependent metalloenzymes that belong to β- and α-classes, respectively. Zinc increases CO_2_ hydration activity of CA1 and CA4, but only CA4 was stimulated by Fe^2+^. In fact, zinc and iron ions are part of the active center in different classes of CAs (Sharma *et al.*, 2009) (Figure S1), and could have stabilization effects on enzyme structure and folding, thus enhancing its activity. Zinc and iron are physiologically relevant cofactors for some CAs (Lionetto *et al.*, 2012). The suppression of CA1 and CA4 activity by EDTA confirmed that the two enzymes are metalloproteins and/or require certain metal ions for its activation. The Hg^2+^ inhibition suggests the presence of a thiol group in the active site of the enzyme, as related to other CAs (Sharma *et al.*, 2009).

The α-Cas, which use Zn^2+^ ions in the active site, are normally monomers and rarely dimers, while β-CAs are normally dimers, tetramers or octamers (Tobal and Balieiro, 2014). In our studies, electrophoretic profile and mass spectrometry analysis suggest that both enzymes, CA1 and CA4, are monomers. These results substantiate functional as well as structural diversity within the CA family.

A multitude of physiologically relevant compounds such amino acids, oligopeptides or small proteins, as well amines (histamines, serotonine and cathecolamines), are known to efficiently activate the catalytic activity of several CAs (Supuran, 2008b; Bertucci *et al.*, 2010). The phenomena is now well understood for the α-class, but not fully elucidated for enzymes belonging to other classes, such as β and γ CAs. The amino acids arginine, tryptophan, histidine and lysine enhance residual activity of CA1 and CA4. The presence of amino acids may increase the number of proton release resulting in an enhancement of *kcat*, with no influence on *Km*. Similar results were observed in CAs encoded by the *Nce103* and *CAN2* genes of *C. albicans* and *C. neoformans,* respectively (Innocenti *et al.*, 2010).

The biochemical characterization of enzymes and gene expression data suggest different roles of the CAs in *Paracoccidioides* physiology. Altogether, our data described for the first time the identification, heterologous expression and characterization of *Paracoccidioides* CAs. Different expression of those enzymes in infection sites suggest that CAs could be relevant for fungus adaptation to different microenvironments. Additional studies about the functional analysis of this class of enzymes in the pathogen *Paracoccidioides* are required.

## Supplementary material

The following online material is available for this article:

Figure S1 - Alignment of the deduced amino acid sequences of carbonic anhydrases.

Table S1 - Oligonucleotide primers used in quantitative real-time PCR and PCR.
