# Supplementary material for: Molecular and biochemical characterization of carbonic anhydrases of Paracoccidioides
Source: Genet Mol Biol. 2016 Jul 25;39(3):416–25. doi: 10.1590/1678-4685-GMB-2015-0213 (PMC5004831; doi:10.1590/1678-4685-GMB-2015-0213)
Supplement: Supplementary file 1 [file 1415-4757-gmb-1678-4685-GMB-2015-0213-Suppl02.pdf]

Table S1 - Oligonucleotide primers used in quantitative real-time PCR and PCR to amplify the complete cDNAs encoding the predicted carbonic anhydrase **(**CAs) of *Paracoccidioides* sp.

**Sequence name**	**Forward Primer (5'→3')**	**Reverse Primer (5'→3')**
qRT-PCR (CA1)	CCTACACAAAGTAGCATCAACA	GAGATATTTGGAGACGTCGG
qRT-PCR (CA2)	CTCCATTGTCATCTCGCAGC	TGCCTGTGACTCTCCACCAA
qRT-PCR (CA3)	GCCAAGCGATACGCGTGTC	CGGTGATAAAAATATACTTCAAGA
qRT-PCR (CA4)	CAGCAGTCCCCCATCGATAT	ATTACCTCCACCGTTGTATTC
Tubulin	ACAGTGCTTGGGAACTATACC	GGGACATATTTGCCACTGCC
XM_002792385.1 (CA1)	GAATTCCATGTTCCGACCCCGCCAG	GTCGACTCACTTCCCCGTCAAATCGT
EU431184.1 (CA4)	GGATCCAAGTCTTTC TTCATCGCAGC	CTCGAGTCAAAAGACAAAAAGAGGTG

Underlined regions correspond to the restriction sites (CA1: *Eco*RI to forward primer and *Sal*I to reverse primer; CA4: *Bam*HI to forward primer and *Xho*I to reverse prim
