# Supplementary material for: Molecular and biochemical characterization of carbonic anhydrases of Paracoccidioides
Source: Genet Mol Biol. 2016 Jul 25;39(3):416–25. doi: 10.1590/1678-4685-GMB-2015-0213 (PMC5004831; doi:10.1590/1678-4685-GMB-2015-0213)
Supplement: Supplementary file 2 [file 1415-4757-gmb-1678-4685-GMB-2015-0213-Suppl01.pdf]

```
CA1             MFRPRQVSTLSTLRTPCTAITTPTQSSINKTSRLVNHQNHLVNKPLPRFPHPCTTRRTIS 60
CA3             ----------------------------------------------------------MA 2
CA2             ------------------------------------------------------------
CA4             MKSFFIAALVFLNAKSVLSSCAHGTHIYPRAAGDVDIDMPKFGYGYDDGPTIWHRLSTAY 60

Ca1             QMAPTDVSKYLQETHERIFENNRKWAAEKAAKDPHFFEKLSMGQNPDYLWIGCSDSRIPA 120
CA3             AGDKNILQDGAEDAYRLALAKNRQWAIKTAEEDPSLFPKLATAQHPEILWIGCSDSRCPE 62
CA2             ---------MVTQIQQNLQDRNAGYASTYNQN------HLAIPPVEKYLVLTCMDARIDP 45
CA4             ALCGSGTQQSPIDIDSTIATMPAGFLSMNIPMQDVKFENLNTTVEVILEGTTMVNGSEFL 120
                            :           :             :*              :.

CA1             EQITGLDPGEAFIHRNIANLVCNTDLNVMSVINYAVRHLHVKHIIVCGHYECGGVKAALT 180
Ca3             TAILGLQPGDVFVHRNIANVIHYNDMSSACVIEYAVVYLKVKHIVLCGHTSCGGIAAALA 122
CA2             SSAFGISLGDAHVIRNAG----ASARDGLRSIVISQQLLGTREILLIKHTGCG-MLTFTN 100
CA4             LKQFHFHTPSEHTLSGEK-YPAEIHMVNLSKDDPKKIVVITLIVQATAGSSISSLGTVIS 179
                     :   . .   .                      : .  :        . : :

CA1             PADLGLLNPWLRNIRDVYRLHEAELDALDENKRYGRLVELNVIEQCRNIIK--------- 231
CA3             NKRLGLLDSWLMPLRRLREQNLYLLNDLNTSEAAEKLAEINVRQGLRTLKE--------- 173
CA2             EQAHALVEKKLVESHRQNGTGAADGGFRVLEALKRDIPDFQPFPLLEEAVR--------- 151
CA4             YIDRITQPSHVVDIPQFNISDVGVAFYILTQALPMHIGDFNVLKGVLKYNSRFLQSPQSA 239
                          :                   .     : :::

CA1             TAA--VQKMYSKNQYPIVHGWVFNIKDG----LLTDLKIDF--EKVLTGIQKIYDLTGK- 282
CA3             NSG--VLDAIQE-RGLKLHGVLYDVGSG----ILRELDVEESVDVVHSRITAFKTLKAEM 226
CA2             DDVRWLSEQVLVTPGIPISGWVYEIETG----KVRRVV---------------------- 185
CA4             NRNVLVAAAGALPSEIVCNGRVATSAAATTFIPRRSLCLFFIFHILVSFLFPSLLFSPLF 299
                     :             * :     .        :

CA1             --
CA3             VH 228
CA2             --
CA4             VF 301
```

Figure S1 - Alignment of the deduced amino acid sequences of carbonic anhydrases. Alignment was observed in several conserved regions of carbonic anhydrases (CAs). Those regions include CAs signature shown in bold, and Zn binding amino acids indicated with a gray rectangle. The amino acid residues conserved in all classes of CAs are shown in an empty rectangle and the highly conserved amino acid residues in β-class of CAs are shown in a dotted rectangle. Asterisks (*) indicates position of complete identity, a colon (:) indicates conserved substitutions and a dot (.) indicates a semi-conserved substitution of amino acid residues.
